# Draft Genome Sequences of Four *Propionibacterium acnes* Strains Isolated from Implant-Related Infections

**DOI:** 10.1128/genomeA.01395-16

**Published:** 2016-12-15

**Authors:** Guillaume Ghislain Aubin, Stanimir Kambarev, Aurélie Guillouzouic, Didier Lepelletier, Pascale Bémer, Stéphane Corvec

**Affiliations:** a Bacteriology and Hygiene Department, Nantes University Hospital, Nantes, France; b EA3826, Laboratory of Clinical and Experimental Therapeutics of Infections, Institut de Recherche en Santé 2, Nantes, France; c Institut de Recherche en Santé de l'Université de Nantes INSERM U892-CNRS 6299 CRCNA Centre de Recherche en Cancérologie Nantes Angers, Université de Nantes, Team 13: Nuclear Oncology Research, Nantes, France; d Institut de Recherche en Santé de l'Université de Nantes INSERM U892-CNRS 6299 CRCNA Centre de Recherche en Cancérologie Nantes Angers, Université de Nantes, Team 2: Clinical and Translational Research in Skin Cancer, Nantes, France

## Abstract

*Propionibacterium acnes* was previously described as a potential implant-related pathogen. Here, we report the draft genome sequence of four *P. acnes* strains, isolated from spine material, hip arthroplasty, and knee arthroplasty infections in France belonging to different sequence types (ST18, ST27, and ST36).

## GENOME ANNOUNCEMENT

*Propionibacterium acnes* is a Gram-positive bacterium constituting a significant part of the human skin microbiota (1). It has been associated with skin diseases such as acne vulgaris or fulminans acne (2). The role of this microorganism in deep and medical device-related infections is underestimated (3). Besides shoulder prosthetic infections, spinal instrumentation infections have been reported (4). Using multilocus sequence typing (MLST) and single-locus sequence typing (SLST) schemes, the *P. acnes* species has been subdivided into five main phylogenetic types: IA1, IA2, IB, IC, II, and III (5, 6). In the context of device-related infections, *P. acnes* antibiotic resistance may be a problem, especially when low- or high-level rifampin resistance is detected (7, 8), as rifampin remains a key drug for eradicating *P. acnes* biofilm infection (9).

Here, we present the draft genome sequences of four *P. acnes* strains (2003-1719, NTS31306190, 2004-10708, and LRY_BL) isolated from patients at Nantes University Hospital and La Roche/Yon Hospital, France, suffering from bone infection.

All *P. acnes* strains were grown overnight at 37°C on Schaedler agar plate (Oxoid, United Kingdom) under an anaerobic atmosphere. Genomic DNA was extracted using a DNeasy blood and tissue kit (Qiagen Gmbh, Germany) as described previously (10). A pair-end library was prepared with a NEBNext Ultra DNA library prep kit for Illumina (NEB) and sequenced (2 × 150 bp) on a MiSeq sequencer (Illumina, USA). *De novo* assembly was performed with Velvet version 1/2/10 and VelvetOptimizer version 2.2.5 (optimal hash value = 127). Contig reordering and annotation were performed with Mauve version 2.3.1 and the NCBI Prokaryotic Genome Automatic Annotation Pipeline (PGAAP), respectively (11, 12). Sequence alignment and comparison were performed with CLC Sequence Viewer version 7.0 and BLAST. Average nucleotide identity (ANI) with the *P. acnes* reference strain KPA171202 was calculated using Oat version 0.91 (13).

The draft genome of strain NTS_2003_1719 (GenBank accession no. MAVU00000000) contains 2,373 genes, 2,320 coding sequences (CDSs), 46 tRNAs, 3 rRNAs, and 4 noncoding RNAs, with an OrthoANI value of 99.1%; the draft genome of strain NTS_31306190 (accession no. MAUY00000000) contains 2,327 genes, 2,275 CDSs, 45 tRNAs, 3 rRNAs, and 4 noncoding RNAs, with an OrthoANI value of 99.1%; the draft genome of strain NTS_2004_10708 (accession no. MAUW00000000) contains 2,322 genes, 2,270 CDSs, 45 tRNAs, 3 rRNAs, and 4 noncoding RNAs, with an OrthoANI value of 99.0%; the draft genome of strain LRY_BL (accession no. MAUX00000000) contains 2,376 genes, 2,327 CDSs, 45 tRNAs, 0 rRNAs, and 4 noncoding RNAs, with an OrthoANI value of 100.0% (Table 1).

**TABLE 1:** Summary of genome sequencing results in the present study

*P. acnes* strain	Clinical source	Reads (Mb)	Coverage (×)	No. of contigs	Size (bp)	G+C content (%)	OrthoANI value^a^ (%)	Accession no.	BioProject designation	SLST	MLST	Phylotype
NTS_2003_1719	Spine material	3,807,168	186	19	2,535,892	60.1	99.1	MAVU00000000	PRJNA327922	D1	ST27	IB
NTS_31306190	Knee prosthesis	3,301,044	127	20	2,479,585	60.0	99.1	MAUY00000000	PRJNA327858	A1	ST18	IA
NTS_2004_10708	Spine material	2,928,004	131	17	2,478,327	60.1	99.0	MAUW00000000	PRJNA327854	A26	ST18	IA
LRY_BL	Hip prosthesis	2,735,728	49	32	2,534,633	60.0	100.0	MAUX00000000	PRJNA327856	H1	ST36	IB

a OrthoANI value compared to the *P. acnes* reference strain KPA171202.

According to the diversity of *Propionibacterium* spp. on human skin (14), their potential involvement in prosthetic-related infections remains an open question for future research. The genome sequences of these four strains of *P. acnes* will also provide a valuable resource for (comparative) bone cell–*P. acnes* host relationship studies. Indeed, depending on their genetic background, *P. acnes* cells seem to interact differently with the bone cell matrix (G. G. Aubin and S. Corvec, unpublished data). These draft genomes of *P. acnes* will also be used for studying virulence features associated with bone infection, especially hyaluronate lyase (15).

### Accession number(s).

This whole-genome shotgun project has been deposited at DDBJ/EMBL/GenBank under the accession numbers listed in Table 1. The versions described in this paper are in the first versions, under the BioProject designations listed in Table 1.
